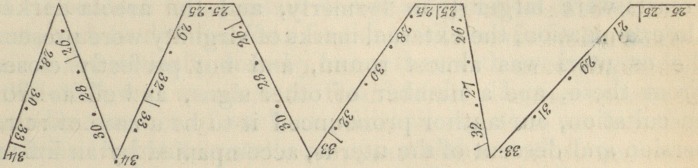# On Obstetric Exploration

**Published:** 1834-04-01

**Authors:** 


*Die Geburtshülfliche Exploration.* Von Dr. ANTON FRIEDRICH HOHL, ausserordent lichem Professor an der Universität zu Halle. *Erster Theil: Das Hören.*—Halle, 1833.

*On Obstetric Exploration.* By Dr. HOHL, Extraordinary Professor in the University of Halle. *Part the First: On Hearing.*—Halle, 1833. 8vo. pp. 314.

IN studying obstetric auscultation, a department of the art of medicine which is confessed on all hands to be very difficult, and is thought by many to be very dubious, it is satisfactory to have the testimony of a witness like the author before us, who is not content with giving his conclusions, but details in the minutest manner the facts upon which they are founded. We have therefore thought it our duty to give a succinct abstract of the work before us, which bears every mark of being the composition of a faithful and zealous observer.

Passing over a good deal of prefatory matter, we come to a chapter entitled "Results of Auscultation in Healthy Women not Pregnant, in Lying-in Women, and in some diseased States of the Abdomen." In some women, says our author, nothing is to be heard but the rolling of gaseous or other fluids; in others, (but they are a minority,) a pulsation is to be heard at the summit of the abdomen, belonging rather to the heart than to the aorta. There is no *soufflet,* however, but a pulsation isochronous with the beating of the heart; the beats are sharply broken off, and no whirring is to be heard between them. In the right part of the upper region of the abdomen, at the left lobe of the liver, a beating may also be heard: this belongs to the tripus Halleri. At the lower and left side of the abdomen another pulsation may be perceived, accompanied by a soufflet: this may proceed from the left iliac artery. It is probably caused by the left iliac artery lying under the vein of the same name. The sound is not heard on the right side.

The flow of the catamenia produces no change in these sounds, nor is any alteration caused by pressing the abdominal parietes.

In lying-in women the above-mentioned pulsations can be perceived, but there is another one in addition. When the uterus is very high up, or even if it is in its ordinary situation, but has not yet perfectly contracted; and there are strong after-pains, a faint humming sound can be heard in that part of the uterus where there was a strong soufflet during pregnancy and parturition; this is almost imperceptibly increased at each beat of the pulse, and follows each pulsation of the heart for an instant.

When there is disease in the abdomen, these rules of course no longer hold good.

When, for instance, the liver was very much enlarged, Dr. Hohl found a pulsation in the right hypochondrium isochronous with the beating of the heart, and the intervals between each beat were filled up with a faint, uniform, obscure soufflet. In a case of fungus medullaris, filling up the whole epigastric region, several mesenteric glands were much enlarged; there was a great accumulation of fæces from obstinate costiveness, and the abdomen was very tense. Here Dr. Hohl was not able to hear the sounds above described; for, to use his own words, "es herrschte eine Todtenstille in der Todbringenden Höhle:" *i.e.* the stillness of death reigned in the death-producing cavity.

In a case of degeneration of the left ovarium, which was taken for pregnancy, our author was unable to hear the pulsation of the iliac arteries. The pulsation of the heart and the cœliac artery could be heard, but not so distinctly as in healthy persons.

In ascites accompanied by tympanites, when our author applied the stethoscope, and gently struck the opposite side, a sound was produced not unlike that of a distant drum. If the abdomen is struck more forcibly, the fluctuation of the water causes a sound something like snoring. The normal sounds are either not heard at all, or there is merely a faint pulsation of the iliac artery.

In aneurism of the aorta there is a strong pulsation and a soufflet. This soufflet, however, is monotonous, and especially perceptible on the left side.

We now arrive at the phenomena which occur in the auscultation of pregnant women.

There are two pulsations peculiar to the gravid state: the one is isochronous with the woman's pulse, the other is much quicker. The first, while it resembles the ordinary pulse in frequency, differs from it in the quality of sound; the latter, on the contrary, has more resemblance to an adult's pulse, excepting in its frequency. It is dicrotic, or doubly beating, and one may count 216, 240, 244, 256, 260, 268, 272, very often 280, not unfrequently 320, 326, nay even 350 single beats; or, what is the same thing, 108, 120, 122, 128, 130, 134, 136, 140, 160, 163, or 175 double beats. This is the fœtal pulse, and its presence or absence determines the life or death of the fœtus.

The isochronous, or placental pulsation, is usually to be heard at the upper and back part of the uterus, especially in first pregnancies. The dicrotic pulse is generally heard towards the left side of the uterus. The placental pulsation has never been heard by Dr. Hohl before the fourth month: it is then much diffused, but in the fifth month becomes concentrated in a single spot. As pregnancy advances, it often becomes weaker and weaker, but never disappears entirely. Nor has our author ever heard the dicrotic pulse before the fourth month; even in the fifth it is weak, and does not become very plain till about the end of the sixth month. In some cases Dr. Hohl could not hear it a short time before the commencement of labour, and in others it disappeared earlier, and in one case it was imperceptible for four weeks before delivery.

The placental pulsation is isochronous, as has been already stated, with the mother's pulse, while the dicrotic pulse is uninfluenced by it.—Our author gives a number of cases to prove this, but we shall content ourselves with quoting the first two.

1. The radial pulse of a pregnant woman was 67 in the horizontal position, 74 when she sat up, and 94 when she stood. The same alterations took place in the placental pulsation, but the dicrotic pulse remained without variation at 140 double beats.

2. The radial pulse of another pregnant woman was 104 when she stood, from 88 to 90 when she sat, and 82 in a horizontal posture. The placental pulsation participated in these changes, but the dicrotic pulse always remained at 132 double beats.

Dr. Hohl, however, is of opinion that the temperature of the mother exercises an influence on the fœtal pulse, the beats being more numerous in proportion to the increase of the heat; and he inserts a long table of observations to prove this. Moreover, when the function of respiration is injured, and the arterialization of the blood is in consequence diminished, the dicrotic pulse loses in frequency. This is especially the case in the earlier months of pregnancy,—that is to say, from the fifth to the eighth month. And hence the diseases of the mother will affect the dicrotic pulse, or not, according to their nature. Thus, in cases of rheumatic fever, or of rheumatism affecting the muscles of the head, abdomen, or extremities, no alteration was perceptible in the dicrotic pulse; but, where the muscles of the chest were affected, or when it was a case of pleurisy; in short, when respiration was encroached upon, this pulse became feebler and slower. The following case is a remarkable one:

A woman of disordered mind had made attempts to strangle herself, so that blood had flowed from her mouth and nose, and the sugillation caused by the cord was visible on her neck for days afterwards; the same night she threw herself into the water, and was restored to sensation with some difficulty. In this case auscultation was the means of consoling the mother, by affording the certain assurance that the child was still alive.

When the stethoscope is applied during labour, a great variety of tone is detected in the placental pulsation: it is stronger, and this increase of energy is especially perceived just before the commencement of a pain. This does not augment the frequency of the dicrotic pulse, nor is this altered by the increased temperature of the woman during labour. Thus, Dr. Hohl found that in several women, between the first stage of labour and the end of the third, the temperature rose to 32° of Reaumur (= 104° of Fahr.), and their pulse became 116, 120, or 124; but the dicrotic pulse kept steadily at 276. In others, in whom the temperature during the third stage of labour was 31° of Reaumur (= 101¾ of Fahr.) and the pulse 112 or 116, the dicrotic pulse was from 280 to 288. The placental pulsation varies with the pains, attains its acme with them, and diminishes in frequency as they gradually go off: and thus the placental pulsation, or, what comes to the same thing, the radial pulse, denotes the character of the pains by its regularity or irregularity, by its attaining or not attaining the desirable frequency, and so on.

Dr. Hohl gives numerous examples of the character of the pains, as indicated by the pulse. The following was an instance of regular pains, and the numbers denote the beats of the pulse, and the placental pulsation, in each quarter of a minute.

C. Albrecht. At the beginning of the second stage of labour: 27. 27. 27. 27. | 27. 28. 28. 30. | 31. 33. 31. 31. | 30. 28. 27. 27. | 27. 28. 30. 32. | 32. 33. 31. 30. | 30. 30. 28. 27. | 27. 27. 27. 27. | 27. 27. 27. 27. | 27. 28. 30. 33. | 31. 28. 27. 27. | 27. 27. 27. 27.—27. 27. 27. 27. | 27. 26. 26. 27. | 27. 27. 27. 26. | 26. 27. 27. 27. | 29. 30. 32. 32. | 30. 28. 27. 27. | &c.

In the middle of the second stage of labour: 27. 27. 27. 27. | 27. 29. 29. 30. | 31. 31. 29. 27. | 27. 27. 27. 27. | 27. 27. 27. 27. | 27. 27. 27. 28. | 29. 30. 31. 31. | 32. 29. 28. 27. | 27. 27. 28. 29. | 27. 27. 27. 28.—28. 28. 29. 30. | 31. 33. 29. 27. | 27. 27. &c.

At the end of the second stage: 28. 28. 29. 30 | 31. 28. 27. 27. | 28. 29. 30. 30. | 28. 28. 27. 27. | 27. 29. 30. 31. | 33. 29. 28. 27.

The pains were regular, but did not follow one another too rapidly, and they lasted for a tolerable time. As long as the pulse was only 27 in a quarter of a minute, the woman was perfectly quiet, and she was tranquil even when the number was 28. At this point the moaning began; at 28 the bag of membranes was slightly advanced, but did not become quite tense till the pulse was 31 or 33 in the quarter of a minute.

We cannot afford room to quote the other cases, but will just mention that Dr. Hohl has constructed some diagrams of very quaint appearance, to shew the pulsations accompanying regular and irregular pains. The following diagram, for instance, exhibits the state of the pulse when the pains are too rapid and stormy, and allow the woman no repose:

Dr. Hohl speaks favourably of the secale cornutum: he finds that, after a few doses, of ten grains each, the placental pulsation is increased both in strength and frequency. The secale, however, must not be old, and must have been well kept.

Dr. Hohl deduces the facts that the *soufflet* belongs to the placental pulsation, and the dicrotic pulse to the fœtus, from a great number of facts and arguments. Thus, to confine ourselves to the former point, the soufflet is never heard in those who are not pregnant, and always heard in those who are; it is generally on the right side, rarely on the left, and still more rarely at the lower part of the uterus; thus following the more usual positions of the placenta. When, however, the placenta presents, the pulsation is heard at the lower part of the uterus; but it is fainter in this case, as this part of the womb is less strong, and so likewise are the vessels belonging to it. This is a very interesting part of Dr. Hohl's work, but to give an analysis of it would lead us beyond all reasonable limits, and we must therefore content ourselves with referring our readers to the original, p. 141-181.

We now come to our author's account of the advantages to be derived from auscultation in the practice of midwifery. The first point achieved by auscultation is the determining cases of doubtful pregnancy. In discussing the uncertainty of the ordinary signs, Dr. Hohl narrates a very remarkable case, which we shall endeavour to abridge. A servant girl complained of morning sickness, want of appetite, lassitude, and absence of the catamenia. A physician was called in, who did not examine her, as he was quite satisfied that she was pregnant, particularly as the breasts had now increased in size, and become painful. Additional symptoms soon made their appearance; she complained of bearing down, and shooting pains in the lower part of the pelvis. The physician now made an examination, and, finding the abdomen distended, the vulva like that of a pregnant woman, the os uteri round, and the uterus larger, he again affirmed it to be a case of pregnancy. The girl, being dismissed from her place, was brought to Dr. Hohl, and confessed that the breasts were larger than formerly, and the areola darker. On examination, the external marks of virginity were present; the os uteri was almost round, and not perfectly closed. From these, and a number of other signs, as well as from auscultation, our author pronounced it to be a case of retroversion and descent of the uterus, accompanied by an inflammatory state, with swelling, and commencing induration.

The treatment consisted in cold injections, and the following powder at night: ℞. Opii, gr. ss.; Camphoræ, gr. ij.; Potassæ Nitr., gr. iij.; Sacch. albi, ℈ss. In a week the patient was considerably relieved, and went back to her former situation. The catamenia now returned, and no one ventured to talk of pregnancy.

Dr. Hohl mentions a case which occurred in 1824, in which Lenormand, by means of the stethoscope, detected a pregnancy, which up to the seventh month was supposed to be a scirrhus of the right ovary.

The next point to be ascertained by the stethoscope is the number of children contained in the uterus: when there are twins, the placental pulsation is far more diffused than usual, and when there are triplets, the sound extends over a still greater space. Dr. Hohl has stethoscopized one case of triplets, and observes that he could tell that there were more than two fœtuses, but could not tell exactly how many; just as, if we listen to the ticking of two watches, we can tell that there are two, but, if a third is added, we can distinguish that there are more than two, but not how many more.

Auscultation is also capable of distinguishing extra-uterine pregnancy. This may be of two kinds. The first is when it exists in the ovarium, the fallopian tube, or the substance of the uterus; the second is when its seat is in the abdomen. But, supposing it to belong to the first class, auscultation will not farther inform us to which subdivision it must be referred.

The existence of pregnancy in the substance of the uterus has been attested by Schmitt, Hederich, Albers, Carus, and Breschet, (*Medico-Chirurg. Transact.*, vol. xiii. part i. p. 33); and some light seems to be thrown upon its progress by a case given by Baudelocque. A woman, who had never had children, died of pleuro-pneumonia in her fifty-third year, and he found a canal in the substance of the uterus, which communicated above with the right fallopian tube, and below with the neck of the uterus. As a placenta is formed in cases of extra-uterine pregnancy, they will be discovered by the usual soufflet being heard in an unusual situation.

As the accoucheur improves in obstetric auscultation, he will be enabled to ascertain the exact position of the fœtal heart, and to deduce from it the position of the head, &c. This is theoretically done by our author with great ingenuity, p. 236-252; but we must confess that the diagnosis seems to us to be a matter of such exquisite nicety, that we fear that centuries of obstetric stethoscopy, or rather cœloscopy, must pass away, before these refinements can be relied upon in practice.

It has always been a desideratum to determine the life or death of the fœtus during childbirth, chiefly of course with reference to the dreadful operation of embryotomy. The occasional fallacy of the ordinary tests of the death of the fœtus is known to every one; perhaps auscultation may furnish a better diagnostic sign. Dr. Hohl lays down the following rule: The fœtus is dead, if the placental pulsation is very faint, or altogether inaudible; if the beating of the fœtal heart cannot be heard in any part of the abdomen, not even when the pregnant or parturient woman is placed in every possible position; and if a deep stillness reigns in the lifeless uterus. Dr. Hohl then considers auscultation in reference to some operations; namely, the bringing on premature delivery, perforation, the Cæsarean operation, turning, and the application of the forceps. He then has a few remarks on the advantages to be derived from auscultation in the treatment of the placenta: when it is retained, the soufflet will point out its exact situation. Dr. Hohl has injected aqua oxymuriatica (solution of chlorine) into the uterus to induce its contraction in cases of hemorrhage and retained placenta. This injection must, we fear, be a very dangerous one; it can hardly be justifiable to introduce a powerful stimulant into the uterus at a time when it is already so prone to inflammation.

Dr. Hohl concludes with some observations on the use of auscultation in cases where the newborn child is apparently dead.

The present work is to be followed by two more, on other branches of obstetrical exploration. Should they be equal to the one of which we have just given an imperfect sketch, they will be alike useful to the profession, and honourable to their distinguished author.

## Figures and Tables

**Figure f1:**